# Tight control – decision-making during T cell–vascular endothelial cell interaction

**DOI:** 10.3389/fimmu.2012.00279

**Published:** 2012-08-27

**Authors:** Sonja Firner, Lucas Onder, Veronika Nindl, Burkhard Ludewig

**Affiliations:** Institute of Immunobiology, Kantonsspital St. Gallen, St. GallenSwitzerland

**Keywords:** high endothelial venules, inflammation, transplantation, costimulation

## Abstract

Vascular endothelial cells (ECs) form the inner layer of blood vessels and exert crucial functions during immune reactions including coagulation, inflammation, and regulation of innate immunity. Importantly, ECs can interact with T cells in an antigen-specific, i.e., T cell receptor-dependent manner. In this review, we will discuss EC actions and reactions during acute inflammation and focus on the interaction of T cells with ECs at two vascular sites: the high endothelial venule (HEV) of lymph nodes, and the vascular lesion during transplant vasculopathy (TV). HEVs are characterized by a highly active endothelium that produces chemoattracting factors and expresses adhesion molecules to facilitate transit of lymphocytes into the lymph node (LN) parenchyma. Yet, T cell–EC interaction at this anatomical location results neither in T cell activation nor tolerization. In contrast, the endothelium at sites of chronic inflammation, such as solid organ transplants, can promote T cell activation by upregulation of major histocompatibility complex (MHC) and costimulatory molecules. Importantly, a major function of ECs in inflamed tissues must be the maintenance of vascular integrity including the efficient attenuation of effector T cells that may damage the vascular bed. Thus, antigen-specific T cell–EC interaction is characterized by a tightly controlled balance between immunological ignorance, immune activation, and tolerization.

## INTRODUCTION

The inner layer of blood vessels, the intima, consists of endothelial cells (ECs) that are attached to the basal membrane. The major function of the endothelium is to control the exchange of gas, metabolites, signal-transmitting molecules, and cells between blood and the tissues. Coping with this range of transport functions requires functional and phenotypical diversity. Hence, ECs can appear as fenestrated endothelium in liver sinusoids that permit free exchange of cells, molecules, and metabolites (Crispe, 2009) or as tight vascular endothelium in the central nervous system that forms a part of the blood–brain barrier (Bechmann et al., 2007). Thus, the particular function of an EC strongly depends on its anatomical location. However, the functional repertoire of ECs can be efficiently modulated by inflammatory stimuli including microbial pathogens or their products or inflammatory mediators derived from other cells.

During an immune reaction, ECs regulate coagulation, react to and secrete acute inflammatory mediators, and coordinate trafficking of leukocytes from the blood stream into the tissue (Danese et al., 2007). Since ECs express not only major histocompatibility complex (MHC) class I and II molecules, but also an array of different costimulatory molecules (Pober and Tellides, 2012), direct and antigen-specific interaction with T cells is possible. Given the diverse phenotypes and functions of ECs together with their functional modulation during inflammatory reactions, it is not surprising that the interaction of T cells with ECs can range from activation to tolerization (Pober and Tellides, 2012). A third interaction pattern is referred to as immunological ignorance (Zecher and Lakkis, 2008). To illustrate these different forms of T cell–EC communication involving MHC–T cell receptor (TCR) contact, we will focus at two distinct vascular sites: the high endothelial venule (HEV) of lymph nodes (LNs), and the vascular lesion during chronic transplant rejection. We propose that the major principle underlying the antigen-specific communication of T cells with ECs is the maintenance of vascular integrity, i.e., the tight control over the exchange of fluids, molecules, and cells between blood and the tissues.

## ENDOTHELIAL CELLS DURING ACUTE INFLAMMATION

The principle of tight control over vascular integrity applies as well to the rapid functional adaptation of ECs during acute inflammation. The basal functions of ECs under homeostatic conditions are the regulation of blood flow and vessel permeability (Dejana et al., 2009). A major control mechanism at the resting state is the inhibition of coagulation which is achieved through the expression of an array of inhibitory molecules including thrombomodulin and heparan sulfate proteoglycans (van Hinsbergh, 2012). Blood flow is regulated by nitric oxide synthase 3 (NOS3) in ECs through the production of nitric oxide, a pathway that alters the tone of vascular smooth muscle cells (Gkaliagkousi et al., 2009). Resting ECs do generally not interact with leukocytes or at least minimize the interaction with leukocytes through the low expression of adhesion molecules such as vascular cell-adhesion molecule 1 (VCAM1) and intercellular adhesion molecule 1 (ICAM1) and the sequestration of adhesion molecules and chemokines in special intracellular storage compartments. However, ECs can react efficiently to perturbations and switch from the resting to an activated state during acute inflammation (Danese et al., 2007; Pober and Sessa, 2007; Lemichez et al., 2010).

Infectious agents can trigger EC activation directly by infection resulting in stimulation of ECs by microbial products sensed via pathogen recognition receptors (Paolillo et al., 2012). Such initial triggers lead to the activation of multiple, partially self-amplifying cascades. For example, the EC growth factor angiopoietin-2 primes ECs to higher responsiveness to tumor necrosis factor leading, in turn, to enhanced leukocyte adhesion (Fiedler et al., 2006). A particular feature of EC activation is the swiftness of their reaction which is achieved through the release of adhesion molecules and inflammatory mediators from their intracellular storage and a rapid change in the gene expression profile (Pober and Sessa, 2007). Further amplification of the initial EC activation is achieved through adherence of platelets. Following contact with activated ECs, platelets release immune-activating factors such as CCL5 (Laubli et al., 2009) which further activate the endothelium and help to recruit immune cells. In addition, platelets interact with the activated endothelium through membrane-bound and soluble CD154, the ligand of CD40, thereby mimicking the interaction of T cells with the endothelium (Henn et al., 1998; Buchner et al., 2003). Importantly, ligation of CD40 on ECs by platelet-derived CD154 promotes tissue factor induction and coagulation (Slupsky et al., 1998).

The rapid local activation of ECs through several cascading systems is most likely a key step during systemic infection and helps to contain the pathogen (Lemichez et al., 2010). However, such powerful activation circuits must be controlled to prevent overshooting clotting reactions, excessive leakage of blood fluids into the tissues, or massive neutrophil degranulation. Indeed, EC activation is restricted by particular regulatory factors such as the Down syndrome critical region gene 1 (DSCR1) which is induced by inflammatory mediators including vascular endothelial growth factor (VEGF) or thrombin (Hesser et al., 2004). Lack of DSCR1 results in elevated ICAM1, VCAM1 and E-selectin expression on ECs and renders ECs more susceptible to apoptosis. Consequently, partially unrestrained EC activation in DSCR1-deficient mice is associated with increased lethality under septic conditions (Minami et al., 2009). Thus, attenuation of EC activation – following a first wave of immune-stimulation – is critical to maintain vascular barrier integrity during acute inflammation. EC-specific mechanisms that maintain barrier integrity include the stabilization of vascular endothelial cadherin function through increased association with p120 catenin subsequently leading to increased resistance against cytokine storm-associated vascular damage (London et al., 2010). A further important property of ECs that most likely improves vascular barrier integrity is their constitutively high resistance to apoptosis, even following exposure to inflammatory stimuli (Bannerman et al., 2001). Taken together, during acute inflammation, ECs can switch rapidly from the resting state into an activated, proinflammatory state that is important for the initiation of the global tissue-defense reaction. However, excessive promotion of the potentially self-promoting inflammatory reactions at the vascular wall must be efficiently attenuated to preserve vascular integrity. We will use the example of chronic transplant rejection to illustrate that the maintenance of vascular integrity through attenuation of endothelial damage by negative immune regulation applies also the antigen-specific interactions between T cells and ECs. Before that, however, we will briefly elude to a third interaction pattern between T cells and ECs, namely attachment and transmigration without cognate or limited MHC–TCR interaction.

## EC–T CELL INTERACTION IN HIGH ENDOTHELIAL VENULES

The induction of efficient T cell responses is fostered by the concentration of both antigen and T cells bearing the appropriate TCR in secondary lymphoid organs (SLOs; Junt et al., 2008). To maximize the chance for successful encounter with their antigen, naïve T cells constantly recirculate through different SLOs (Mempel et al., 2006). It is noteworthy that not only the nature of the SLO, e.g., LNs or Peyer’s patches (PPs) critically impinge on EC–T cell interaction, also differences between anatomically distinct LNs results in qualitatively different interaction patterns between T cells and EC (Buettner and Bode, 2011). The ability of lymphocytes to enter LNs and PPs depends on the presence of specialized post-capillary venules. These HEVs are formed by specialized ECs that have been described as paracortical, vascular endothelium containing cuboidal ECs (Anderson and Anderson, 1976). HEV ECs develop a polarized organization with luminal adhesion molecules such as ICAM1 which function as anchors for cells circulating in the blood and expressing the appropriate ligands (Blum and Pabst, 2006). Tethering and rolling of lymphocytes on the HEV endothelium is further enhanced in certain anatomical locations through the expression of particular adhesion molecules such as the mucosal addressin cell adhesion molecule-1 (MAdCAM1). Expression of this mucosal addressin on HEVs in the mesenteric LN and PPs mediates the interaction with α_4_β_7_ integrin on a subset of lymphocytes and facilitates homing of T cells with a more gut-restricted TCR repertoire (Sigmundsdottir and Butcher, 2008). Furthermore, the endothelium of HEVs produces the constitutive chemokines CCL19, CCL21, CXCL12, and CXCL13. These small chemoattractant cytokines bind to G protein-coupled chemokine receptors on lymphocytes and foster thereby T cell migration, activation, and proliferation (Hayasaka et al., 2010). Hence, the endothelium of HEVs facilitates the highly efficient transit of T cells from the blood stream into the LN or PP parenchyma. In other words, HEV ECs are constantly in close contact with naïve T cells and other migrating hematopoietic cells, a feature that is not shared with other ECs.

Endothelial cells arise from endothelial progenitor cells that are recruited from the mesodermal layer and form the large vasculature of the early mammalian embryo. HEV ECs develop together with the LN when lymphatic endothelial progenitors leave the cardinal vein and form the lymph sac, the primordial tissue of the lymphatic system (van de Pavert and Mebius, 2010; Domigan and Iruela-Arispe, 2012). During LN development, mesenchymal and hematopoietic cell-derived signals initiate chemokine expression and LN growth (van de Pavert and Mebius, 2010). Since lymphotoxin beta receptor (LTβR)-deficient mice completely lack peripheral LNs, it has been suggested that LTβR-signaling in mesenchymal organizer cells is crucial for LN development (Roozendaal and Mebius, 2011). Indeed, hematopoietic cell-derived lymphotoxin induces the expression of cytokines and chemokines in non-hematopoietic stromal cells (Ansel et al., 2000). Thus, HEV ECs develop in an environment of highly active signal exchange between hematopoietic and non-hematopoietic cells. Hence the extensive interaction of HEV ECs with T cells may function not only via adhesion molecules or chemokine–chemokine receptor pairs, but also via antigen-specific TCR–MHC contact.

Antigen presentation and activation of T cells is a well-controlled process that relies to a large extent on a division of labor between different myeloid cell subsets (Turley et al., 2010). It is possible that a similar specialization in the display of self-antigens for the tolerization of autoreactive T cells can be assigned to different stromal cell subsets. Indeed, several studies suggest that stromal cells such as lymphatic ECs or T cell zone fibroblastic reticular cells (FRC) in LNs express peripheral tissue antigens (PTAs) in order to mediate peripheral tolerance to autoreactive T cells (Gardner et al., 2008; Cohen et al., 2010; Fletcher et al., 2010). However, it is unlikely that HEV ECs can perform a similar task because ubiquitous expression of an antigen in ECs driven by the Tie2 promoter does neither lead to activation nor tolerization of antigen-specific CD8^+^ T cells (Bolinger et al., 2008). Likewise, FRCs can present viral antigen during systemic infection with the non-cytopathic lymphocytic choriomeningitis virus leading to the elimination of these stromal cells by antiviral CD8^+^ effector T cells (Mueller et al., 2007; Scandella et al., 2008). However, HEV ECs seem not to be affected by immunopathological CD8^+^ T cells during this viral infection. On the contrary, stimulation of HEV ECs via LTβR through B cell-derived lymphotoxin was found to be very important for the adaptation of the LN, i.e., for efficient LN remodeling (Kumar et al., 2010). Taken together, in a homeostatic LN, T cells appear not to communicate with HEV ECs in an antigen-specific manner, i.e., the interaction pattern of immunological ignorance is predominant. Whether HEV ECs are specifically protected from immunopathological T cell attack or whether they remain immunologically ignored even during systemic viral infection remains to be determined. Clearly, ECs do not remain immunologically ignored during transplant rejection.

## ANTIGEN PRESENTATION BY ECs DURING CHRONIC TRANSPLANT REJECTION

Graft rejection after solid organ transplantation is characterized by the recognition of the donor tissue as foreign and subsequent attack by the host immune system. The immunological reaction can be directed against parenchymal cells or cells of the vascular system. Acute graft rejection (in the absence of immunosuppression) occurs usually 1–2 weeks following transplantation. These grafts characteristically contain dense leukocyte infiltrates in the parenchyma and show extensive vessel thrombosis. Chronic immunological reactions of the host against the graft that may occur despite immunosuppression, can be directed against the parenchyma resulting in progressive fibrotic replacement of graft tissue (Libby and Pober, 2001). However, more frequent is the chronic immune-mediated damage of blood vessels. Despite advances in immunosuppressive therapies for acute allograft rejection, successful long-term survival of transplanted solid organs is still hampered by late graft failure. Chronic graft rejection is caused to a large extent by host-anti-graft immune responses against the graft vasculature leading to transplant vasculopathy (TV; Cailhier et al., 2006). Since ECs of the transplanted organ are the first graft cells encountered by the host immune system and ECs are preserved in long-term allografts (Al-Lamki et al., 2008), it is most likely that T cell responses against ECs crucially contribute to the process of chronic vascular rejection (Libby and Pober, 2001).

It has been demonstrated that ECs can act as antigen-presenting cells (APC) to CD8^+^ T cells mainly via the direct pathway (i.e., recognition of allo-MHC complexes). However, *in vitro* experiments suggest that ECs directly stimulate mainly pre-activated memory but not naïve CD8^+^ T cells (Dengler and Pober, 2000). EC-specific CD8^+^ T cells have been shown to exist *in vivo* and are able to mediate significant EC damage in human graft-versus-host disease (Biedermann et al., 2002). Furthermore, it has been demonstrated in a transgenic mouse model that MHC class I expression on non-hematopoietic cells of the graft is sufficient to initiate CD8^+^ T cell activation and acute allograft rejection (Kreisel et al., 2002). These results from a TCR-transgenic system have been interpreted as evidence for the direct activation of CD8^+^ T cells by ECs outside of SLOs. However, direct recognition of allo-MHC complexes by the highly frequent alloreactive T cells can only occur under conditions of MHC disparity, i.e., in allogeneic mixed-lymphocytes reactions *in vitro* or following transplantation of MHC mismatched organs.

**FIGURE 1:**
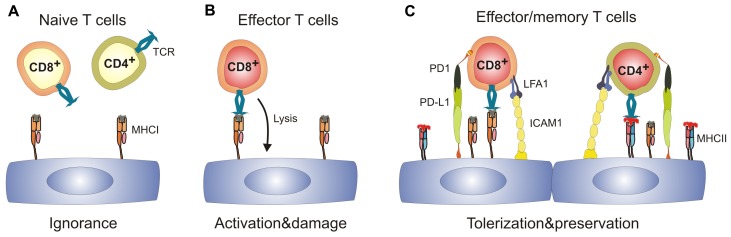
**Spectrum of antigen-specific T cell–EC interactions.(A)** naïve T cells immunologically ignore resting ECs which express MHC class I, but are MHC II^low^ or MHC II^-^. **(B)** Effector CD8^+^ T cells can recognize cognate antigen on ECs leading to EC activation and may potentially damage the vascular bed. **(C)** Activated ECs upregulate expression of MHC II, adhesion molecules and ligands of T cell co-inhibitory molecules. Engagement of co-inhibitory molecules can lead to tolerization of both CD8^+^ and CD4^+^ T cells and hence preserves the integrity of the EC layer. ICAM1, intercellular adhesion molecule 1; LFA1, leukocyte function-associated antigen 1; MHC I/II, major histocompatibility complex I/II; PD-1, programmed cell death 1; PD-L1, programmed cell death ligand 1; TCR, T cell receptor.

Whereas T cell precursor frequencies against the “major” alloantigens, i.e., directly recognized MHC molecules, are in a range of 0.1–10%, T cell precursor frequencies against minor histocompatibility antigens (mHAg) are low (Heeger, 2003). It is noteworthy that due to the almost complete MHC matching procedures in transplantation medicine (Cecka, 2010), transplant rejection is mainly driven by T cell reactions against mHAg (Spencer et al., 2010). Hence, EC–T cell interaction during TV is characterized by low T cell precursor frequencies, whereby the T cells most likely recognize antigen presented by ECs. To model this situation experimentally, expression of a model antigen can be directed to vascular ECs using the Tie2 promoter (Tie2-LacZ mice) (Schlaeger et al., 1997). Using this EC-specific mHAg expression system in combination of mHAg-specific TCR transgenic T cells, it could be shown that mhAg presentation by EC does neither precipitate T cell activation nor tolerization (Bolinger et al., 2008), i.e., tolerizing effects on CD8^+^ T cells were not observed, although resting mhAg-presenting ECs in Tie2-LacZ mice provided signal 1 (i.e., antigen) in the absence of signal 2 (i.e., costimulation). Hence, in the absence of appropriate stimulation, naïve CD8^+^ T cells ignore their antigen presented solely on ECs (**Figure 1A**). In principle, it is possible that ECs possess an impaired capacity to present immunodominant peptides (Kummer et al., 2005) and therefore fail to interact with naive CD8^+^ T cells. However, once appropriately activated, T cells can form invadosome-like protrusions that permit probing of the MHC:peptide complexes expressed on ECs (Carman et al., 2007; Sage et al., 2012).

The presence of SLOs is critical for the generation of transplant-specific T cells (Lakkis et al., 2000). Furthermore, priming of mHAg-specific CD8^+^ T cells has been shown to be strictly dependent on cross-presenting CD11c^+^ DCs (Bolinger et al., 2008; Wang et al., 2011). In addition, other myeloid cells can enter the graft to sample antigen and return to the local LN to initiate T cell responses (Celli et al., 2011). Thus, ECs in transplanted organs expressing a particular antigen can become targets for CD8^+^ effector T cells (**Figure1B**) once professional APCs have presented the peptide within SLOs. As a consequence, grafts can develop a vascular inflammatory disease with neointima formation and vascular occlusion, the pathological signs of chronic vascular rejection (Bolinger et al., 2010).

However, antigen recognition on ECs does not necessarily lead to aggression. ECs could negatively regulate immune responses by utilizing co-inhibitory receptors such as Herpes simplex entry mediator (HVEM; Murphy and Murphy, 2010). Clearly, programmed cell death ligand-1 (PD-L1) expression on mHAg-presenting ECs is strongly upregulated during inflammation (Bolinger et al., 2010). Importantly, PD-L1 expression on ECs is regulated to a large extent via the IFN-γ receptor (Grabie et al., 2007; Bolinger et al., 2010) and the efficacy of PD-1-dependent CD8^+^ T cell down-tuning correlates with the levels of systemic IFN-γ (Bolinger et al., 2010). As a consequence, upregulation of negative regulatory factors such as PD-L1 on ECs provides a potent negative feedback for EC-specific CD8^+^ T cells and thereby reduces vascular pathology (**Figure 1C**; Bolinger et al., 2010). Importantly, this mechanism may not only operate in chronic transplant rejection, but may also limit endothelial destruction and, thus fatal parenchymal damage during viral infection (Iwai et al., 2003; Barber et al., 2006). Taken together, expression of co-inhibitory molecules on ECs during inflammatory processes appears to be a central regulatory step in the control of EC-specific CD8^+^ T cell responses and hence, in the promotion of shielding tissues from T cell-mediated damage.

## CONCLUDING REMARKS

Maintenance of vascular integrity during inflammation, i.e., securing the barrier function of the endothelium, represents an important challenge for the cooperation between the immune and the vascular system. Tight control over the exchange of fluids, molecules, and cells between blood and tissues during antigen-specific EC–T cell interaction is achieved through different mechanisms. Importantly, naïve T cells ignore their cognate antigen on ECs and only adequately activated T cells can recognize their antigen on ECs and subsequently exert their effector function. Since recognition of tissues by CD8^+^ effector T cells can precipitate severe immunopathological consequences, potent tissue-protective mechanisms must be activated during the antigen-specific interaction of these cell types. Hence, the ligation of PD-1 or the HVEM-receptor BTLA on EC-specific effector T cells represents an attractive therapeutic target to avoid excessive EC damage during inflammation. Furthermore, cell type-specific signal transduction pathways downstream of the IFN-γ receptor in ECs (Miura et al., 2006) may harbor specific targets that could permit stimulation of peripheral inhibitory signals. Clearly, further research is warranted to better understand how proinflammatory stimuli can be translated locally into anti-inflammatory signals for the benefit of vascular and tissue integrity.

## Conflict of Interest Statement

The authors declare that the research was conducted in the absence of any commercial or financial relationships that could be construed as a potential conflict of interest.

## Abbreviations

FRC: fibroblastic reticular cell
LN: lymph node
mHAg: minor histocompatibility antigen
SLO: secondary lymphoid organ
